# Hypoglycemic Hemiparesis Presenting as a Stroke Mimic: A Case Report

**DOI:** 10.7759/cureus.84222

**Published:** 2025-05-16

**Authors:** Joel Collins, James Espinosa, James Lee, Alan Lucerna

**Affiliations:** 1 Emergency Medicine, Jefferson Health, Stratford, USA

**Keywords:** acute hemiplegia due to hypoglycemia, hemiparesis due to hypoglycemia, hypoglycemic hemiparesis, hypoglycemic stroke mimic, stroke mimics

## Abstract

Here, we present the case of a 47-year-old male with a history of diabetes mellitus (DM), who was brought to the emergency department (ED) with right-sided hemiparesis and slurred speech. His blood sugar was 37 mg/dL. An intravenous line was established, and the patient was administered intravenous dextrose. Within five minutes, his symptoms had significantly improved and, by 15 minutes, had resolved completely. A CT head and CT angiogram of the head and neck were within normal limits. Basic laboratory tests were within normal limits. The initial neurology service consult concluded that his diagnosis was hypoglycemic hemiplegia. The patient was admitted to the hospital for evaluation of blood sugar and for an MRI brain. The patient required one more dose of dextrose two hours after arrival for a blood sugar of 60 mg/dL. The MRI brain was normal. This case illustrates that hypoglycemia can present with stroke-like symptoms. In our case, the etiology of the hypoglycemia itself was associated with missed meals and a relatively excessive medication regimen for diabetes. It is for such cases as the one presented that stroke protocols emphasize early assessment of the blood glucose level.

## Introduction

Our patient presented with right upper extremity and right lower extremity weakness of a one-hour duration, which triggered a stroke evaluation. Ultimately, a diagnosis of hypoglycemia hemiplegia was made. This case illustrates that hypoglycemia can present with stroke-like symptoms. Non-stroke diagnoses that present with stroke-like symptoms are referred to in the medical literature as “stroke mimics,” and such presentations can imitate acute ischemic in up to 30% of suspected stroke cases [1,2]. One review suggests that the rate of stroke mimics may be even higher than 30% and discusses two scoring systems to reduce over-diagnosis. The most common causes of stroke mimics are psychogenic causes, seizures, migraine, metabolic causes such as hypoglycemia, infection, central tumors, hypertensive encephalopathy, and the posterior reversible encephalopathy syndrome. [3,4].

This case report was presented in poster form at the Rowan-Virtua SOM Research Day, Stratford, NJ, on May 1, 2025.

## Case presentation

A 47-year-old male with a history of diabetes mellitus (DM) was brought to the emergency department (ED) by his wife by private vehicle, with a history of the sudden onset of right arm and right leg weakness while beginning to eat his lunch. His medications included metformin 1000 mg twice a day and glipizide 5 mg once a day with breakfast. His wife stated that he had not been feeling well for the prior three days and had eaten little in the last 24 hours. He did not perform regular home monitoring of his blood sugar levels. He took his morning dose of metformin and glipizide but did not eat breakfast. He had some confusion, according to his wife, who helped him to his car with the assistance of a neighbor.

On arrival at the ED, the patient was brought into the treatment area in a wheelchair with the assistance of the ED staff. His speech showed mild to moderate dysarthria and slurring, but could be understood. He was awake and followed commands. There was no facial asymmetry. His right arm and leg showed weakness, with some effort against gravity. His NIH stroke scale score was five. The time frame from the onset of symptoms to arrival at the ED was approximately one hour. A stroke alert was called simultaneously with a bedside glucose check. His blood sugar was 37 mg/dL (Table 1). 

**Table 1:** Emergency department laboratory values. Abbreviations: BUN (blood urea nitrogen), PT (protime),  PTT (partial thromboplastin time),  INR (international normalized ratio),   K/uL (1000 per microliter), g/dL(grams per deciliter), mEq/L (milliequivalents per liter), mg/dL (milligrams per deciliter), mcg/ml (micrograms per milliliter), cells/HPF (cells per high powered field), mmol/L (millimoles per liter)

Laboratory results in the emergency department	Result	Normal range	Units
White blood cell count	10.2	4.0-11.0	K/uL
Hemoglobin	12.6	10.6-15.6	g/dL
Platelet count	210.0	150-400	K/uL
Sodium	138	135-154	mEq/L
Potassium	3.6	3.5-5	mEq/L
BUN	19.0	5 to 20	mg/dL
Creatinine	1.10	0.6-1.2	mg/dL
Glucose	37	70-100	mg/dL
Calcium	9.1	8.5-10.5	mg/dL
Chloride	102	95-105	mEq/L
Bicarbonate	27	23-29	mEq/L
Lactate	1.0	0.5 to 2.2	mmol/L
Magnesium	1.8	1.7-2.2	mg/dL
PT	11.0	11 to 13	sec
PTT	33.0	25 to 35	sec
INR	1.0	0.8 to 1.1	NA
Urine color	yellow	yellow	NA
Urine clarity	clear	clear	NA
Urine specific gravity	1.016	1.005-1.030	NA
Urine pH	7	5 to 7.5	NA
Urine glucose	negative	negative	NA
Urine protein	negative	negative	NA
Urine bilirubin	negative	negative	NA
Urine urobilinogen	negative	negative	NA
Urine ketones	negative	negative	NA
Urine blood	negative	negative	NA
Urine white cells	negative	0-5/HPF	cells/HPF
Urine red cells	negative	0-5/HPF	cells/HPF
Urine nitrite	negative	negative	NA
Urine leukocyte esterase	negative	negative	NA

An intravenous line was established quickly, and the patient was administered intravenous dextrose. Within five minutes, his symptoms had significantly improved and, by 15 minutes, had resolved completely.

A CT head and CT angiogram of the head and neck were within normal limits. Basic laboratory tests were within normal limits. The initial neurology service consult noted that his diagnosis was hypoglycemic hemiplegia. The patient was admitted to the hospital for evaluation of blood sugar and an MRI brain. The patient required one more dose of dextrose two hours after arrival for a blood sugar of 60 mg/dL. The MRI brain was normal. The neurology service concluded that the diagnosis was hypoglycemic hemiplegia. He was discharged from the hospital in good condition with a decrease in both metformin and glipizide.

## Discussion

The most common neurologic symptoms of hypoglycemia are confusion and personality changes. The most common autonomic symptoms are diaphoresis and tremor. Hemiparesis, however, is a rare indication of hypoglycemia and can be attributed to stroke. A review of hypoglycemic presentations suggested that neurologic symptoms could be divided into depressed sensorium, behavioral change, dizziness, tremor, seizures, and sudden hemiparesis. Sudden hemiparesis was the least common of the categories. Hemiparesis associated with hypoglycemia tends to have a right-sided predominance for unclear reasons. It has been hypothesized that the right-sided predominance may be different due to a difference in metabolism or density of neural tissue between the hemispheres [5,6].

The actual mechanism of hemiparesis associated with hypoglycemia has not been completely clarified. There are several theories. A pre-existing, narrow artery and hypoglycemic-induced vasospasm may be explanatory in some cases of hypoglycemic paresis. Vasospasm can be triggered by hypoglycemia itself and can affect smaller cortical vessels.

Perhaps the most common current hypothesis has to do with regions of the brain with greater vulnerability to hypoglycemia. It is known that the different regions of the brain demonstrate variable responses to such stimuli as intoxicants, metabolic disorders, and hypoxia. Areas of greater metabolic activity in the brain may make some more vulnerable to hypoglycemia. Some cases of hypoglycemic hemiparesis have shown abnormal findings on CT scan, MRI with diffusion-weighted images, and single-photon emission computed tomography (SPECT scan). The most commonly affected areas appear to be the internal capsule and the splenium of the corpus callosum. There are case reports of reversible abnormal findings in the cortical layer, pons, basal ganglia, substantia nigra, hippocampus, thalamus, and hypothalamus [7-9].

In our case, the etiology of the hypoglycemia itself was probably associated with missed meals and a relatively excessive medication regimen for diabetes.

## Conclusions

Here, we present the case of a 47-year-old male with a history of DM, who was brought to the ED with hemiparesis of the right side and slurred speech. His blood sugar was 37 mg/dL. An intravenous line was established quickly, and the patient was administered intravenous dextrose. Within five minutes, his symptoms had significantly improved and, by 15 minutes, had resolved completely. A CT head and CT angiogram of the head and neck were within normal limits, as was a subsequent MRI of the brain. This case illustrates that hypoglycemia can present with stroke-like symptoms. It is for such cases that stroke protocols emphasize early assessment of the blood glucose level.
